# The value of combined examination of serum serum heparin binding protein, tumor necrosis factor alpha, interleukin-6, platelet count, and c-reactive protein in evaluating the condition and prognosis of children with adenovirus pneumonia

**DOI:** 10.3389/fped.2025.1620206

**Published:** 2025-09-03

**Authors:** Guangjun Wang, Jibin Liu, Xuejiao Yu, Shuzhen Cao

**Affiliations:** ^1^ Pediatrics Department, Qingdao Chengyang District People’s Hospital, Qingdao, Shandong, China; ^2^ Pediatrics Department, Anqiu Women and Children’s Hospital, Weifang, Shandong, China

**Keywords:** adenovirus pneumonia, pediatrics, biomarkers, prognostic model, inflammatory response, heparin binding protein

## Abstract

**Background:**

Adenovirus pneumonia presents a significant challenge in pediatric care, with severe children leading to significant morbidity. The study evaluated the combined prognostic value of serum Heparin Binding Protein (HBP), Tumor Necrosis Factor-alpha (TNF-α), Interleukin-6 (IL-6), platelet count (PLT), and C-Reactive Protein (CRP) levels in assessing the condition and prognosis of children with adenovirus pneumonia.

**Methods:**

This retrospective study analyzed children with adenoviral pneumonia at our hospital from March 2019 to December 2024. Serum levels of HBP, TNF-α, IL-6, PLT, and CRP were measured. Participants were divided into non-severe and severe groups, and further into good and poor prognosis groups, based on clinical criteria and complications. Statistical analyses compared biomarker levels across these groups and evaluated their prognostic accuracy using receiver operating characteristic (ROC) curve analysis.

**Results:**

Elevated serum levels of HBP, TNF-α, IL-6, and CRP were significantly associated with severe adenovirus pneumonia (*P* < 0.001) in 300 children. Lower PLT was noted in severe children (*P* < 0.001). The composite model exhibited a high predictive accuracy with an AUC of 0.945 for disease severity and 0.940 for prognosis.

**Conclusion:**

The combination of serum HBP, TNF-α, IL-6, PLT, and CRP effectively predicted disease severity and prognosis in pediatric adenovirus pneumonia.

## 1 Introduction

Pneumonia caused by Adenovirus is a significant cause of pediatric respiratory illness, characterized by its potential for severe morbidity and the challenge it presents to effective clinical management (1). This condition is particularly concerning in children, as their developing immune systems render them more susceptible to respiratory pathogens, often leading to severe sequelae (2). Adenovirus, a non—enveloped double—stranded DNA virus, is well—known for its ability to persist in the environment and within host cells (3). This property contributes to the frequent outbreaks of respiratory illness among young populations globally.

Despite notable advancements in antiviral therapies and supportive care in recent years, accurately predicting the severity and prognosis of adenovirus pneumonia in children remains a formidable challenge (4). This is primarily because current diagnostic protocols for adenovirus pneumonia predominantly rely on clinical evaluations and nucleic acid—based tests, such as quantitative polymerase chain reaction (qPCR), to confirm the presence of the virus (5). However, these methods are limited in that they do not provide comprehensive information regarding disease severity or prognosis (5).

In the pursuit of more effective clinical management, the exploration of biomarkers as tools for clinical assessment has gained significant traction in recent years (6). Biomarkers have the potential to bridge the gap between viral identification and clinical outcomes, offering valuable insights into disease outcomes and patient prognosis. Among the numerous biomarkers investigated, serum heparin—binding protein (HBP), tumor necrosis factor—alpha (TNF—α), interleukin—6 (IL—6), platelet count (PLT), and C—reactive protein (CRP) have emerged as promising candidates. Their roles in the inflammatory cascade and immune response suggest they could be closely associated with the severity and prognosis of adenovirus pneumonia in children (7).

HBP is recognized for its crucial role in modulating vascular permeability and enhancing the inflammatory response. As such, it serves as a reliable marker of acute inflammation, potentially correlating with disease severity. Elevated HBP levels have been consistently associated with worse clinical outcomes in various inflammatory and infectious diseases, highlighting its potential value in the evaluation of pediatric adenovirus pneumonia (8). For instance, in a study by Xiao-Yan Liu et al., it was demonstrated that Compared with the non-severe adenovirus pneumonia group, the severe adenovirus pneumonia group had a significantly higher serum level of HBP (9). Similarly, TNF-α and IL-6 were pivotal cytokines that mediate immune responses and inflammation (10). Their upregulation was well-documented in severe respiratory infections, where they contribute to the recruitment and activation of immune cells, perpetuating the inflammatory milieu and exacerbating tissue damage (11). Research by Yue Hou et al. showed that elevated levels of TNF—α and IL—6 could be helpful to judge the severity of adenovirus pneumonia, which could be used as the objective indexes to evaluate the prognosis of children with severe adenovirus pneumonia (12).

Alongside cytokines, CRP, an acute-phase protein produced by the liver in response to inflammation, offers a practical measure of systemic inflammation (13). Its levels rise in proportion to the intensity of the inflammatory response and were often correlated with disease severity across a spectrum of infectious conditions (14). PLT, though traditionally considered a component of routine hematological evaluations, has gained recognition for its association with infectious disease outcomes (9). Thrombocytopenia in the context of infection was frequently indicative of disease severity, possibly reflecting increased platelet consumption due to inflammation or dissemination intravascular coagulation (15).

The objective of this study was to comprehensively evaluate the combined prognostic value of serum HBP, TNF—α, IL—6, PLT, and CRP in pediatric adenovirus pneumonia. By doing so, we aimed to provide a more accurate and effective means of predicting disease severity and prognosis, ultimately guiding more targeted and personalized clinical management strategies.

## 2 Materials and methods

### 2.1 Study design and ethics statement

This study retrospectively analyzed data from pediatric patients diagnosed with adenovirus pneumonia and treated at our hospital between March 2019 and December 2024. The study was approved by the Ethics Review Board and Ethics Committee of Anqiu Women and Children's Hospital (no ethical number), and complied with the pertinent statements of the Declaration of Helsinki. For this retrospective analysis, informed consent was waived by the Institutional Review Board and Ethics Committee due to the use of de-identified data, ensuring that there was no potential risk or impact on the care of the patients.

### 2.2 Patient selection criteria and grouping basis

#### 2.2.1 Inclusion and exclusion criteria

##### 2.2.1.1 Inclusion criteria

Participants were children aged 0–18 years who were hospitalized and received treatment. They had complete data available for analysis, with adenovirus infection confirmed by PCR within 48 h prior to or following admission. All participants met the diagnostic and therapeutic guidelines for pediatric adenovirus pneumonia.

##### 2.2.1.2 Exclusion criteria

Exclusions applied to those who were discharged and readmitted within 28 days, received antibiotic treatment within 72 h before hospitalization, displayed pulmonary parenchymal or interstitial changes due to other diseases such as lung cancer, tuberculosis, or pulmonary embolism, had autoimmune diseases or were immunocompromised, suffered from infections at other sites, experienced intractable respiratory failure, or had serious comorbidities unrelated to pneumonia, such as intracranial hemorrhage or multiple injuries.

Diagnosis of adenovirus pneumonia was established according to the guidelines for the prevention and control of human adenovirus (HAdV) respiratory infections and diagnostic criteria for pediatric adenovirus pneumonia (16). Detection of adenovirus infection involved collecting nasopharyngeal swabs, sputum, and bronchoalveolar lavage fluid samples. Adenovirus nucleic acid was identified using real-time qPCR (qRT-PCR). A viral load exceeding 10^3^ copies/ml in any of the aforementioned specimen types confirmed adenovirus infection.

#### 2.2.2 Severity grouping

Prior to treatment, the condition of the patients was assessed using the Nelson Textbook of Pediatrics (17), a widely recognized standard reference in pediatric medicine. Patients were categorized into the non-severe group if they did not exhibit severe symptoms. The severe group included patients with any of the following conditions: deterioration of general health, signs of dehydration/refusal to eat, consciousness disorders, cyanosis, rapid breathing (≥50 breaths/min), use of accessory respiratory muscles (grunting, nasal flaring, intercostal retractions), intermittent apnea, or oxygen saturation below 92%.

#### 2.2.3 Post-treatment prognosis grouping

The prognosis following treatment was evaluated based on the presence of complications. Patients without complications formed the good prognosis group. Those with any complication associated with adenovirus pneumonia were classified into the poor prognosis group, following criteria from the Clinical Epidemiology of Adenovirus Pneumonia among Chinese Hospitalized Children (18). Specific complications included: Atelectasis, Pneumothorax, Toxic Encephalopathy, Seizures, Diarrhea, Abdominal Pain, Coagulation Disorders, Electrolyte Imbalances.

### 2.3 Treatment protocol

During hospitalization, all patients received standardized supportive treatment. Supportive care included oxygen therapy through a nasal cannula (Yuwell Medical Equipment Co., Ltd., YUWELL-ND-B, China) with an oxygen flow rate of 2 L/min, continuously monitoring blood oxygen saturation to maintain levels above 95%. Additionally, for severe cases, patients received intravenous immunoglobulin (IVIG) (Hualan Biological Engineering Co., Ltd., 202308L5) at a total dose of 1.0 g/kg/d, once daily, administered over two days. This approach aligns with current guidelines recommending IVIG for its role in modulating inflammation and improving outcomes in severe adenovirus pneumonia (19).

### 2.4 Data collection

Basic demographic information, such as age and height, was recorded in the electronic medical record system prior to patient admission. The duration of fever was calculated by summing the number of days with fever before and after admission. The duration of coughing was recorded following the same method. Additional symptoms were documented based on the last diagnosis before admission. During hospitalization, patients underwent serum and whole blood examinations in the initial phase of treatment.

### 2.5 Whole blood analysis

Blood samples were taken after admission to the hospital and before treatment. In a non-fasting state, 2 ml of venous whole blood was collected from the pediatric patients. Blood analysis was conducted using an automated hematology analyzer (BC-6800Plus, Mindray, China) after ensuring the instrument was regularly calibrated and subjected to daily quality control checks. The analyzer's computer system processed the collected pulse signals to compute complete blood count data, including PLT, white blood cell count (WBC), lymphocyte count, hemoglobin (Hb) levels, and other hematological parameters. The samples were centrifuged at 3,000 rpm (centrifugal radius: 10 cm) for 10 min to separate serum. Serum levels of HBP, TNF-α, IL-6, CRP, procalcitonin (PCT), complement 3 (C3), and complement 4 (C4) were measured using an electrochemiluminescence immunoassay analyzer (Cobas e601, Roche Diagnostics, Switzerland). Serum lactate dehydrogenase (LDH) levels were detected using a biochemical analyzer (BA-800M, Mindray, China).

### 2.6 Statistical analysis

Data analysis was conducted using SPSS version 29.0 (SPSS Inc, Chicago, IL, USA). Categorical data were expressed as [*n*(%)], analyzed by chi-square or Fisher's exact test as appropriate. Continuous variables were tested for normality (Shapiro–Wilk) and compared using *t*-tests (normally distributed). A two-tailed *P*-value of <0.05 was considered statistically significant. Comparisons of HBP, TNF-α, IL-6, PLT, and CRP among the groups were conducted. The diagnostic performance of serum HBP, TNF-α, IL-6, PLT, and CRP combination for pediatric adenovirus pneumonia was assessed by analyzing the area under the curve (AUC) of the receiver operating characteristic (ROC).

## 3 Results

### 3.1 Severity grouping

**Figure 1:**
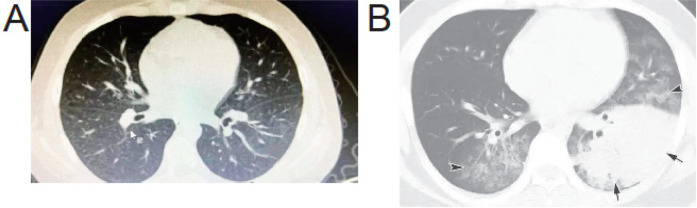
Comparison of lung CT scans between the two patient groups. **(A)** Non-severe Group, **(B)** Severe Group.

The baseline characteristics of 300 pediatric patients diagnosed with adenovirus pneumonia between the Non-severe Group (*n* = 195) and Severe Group (*n* = 105) revealed significant differences in age, with the Non-severe Group having a mean age of 6.65 ± 1.84 years compared to 5.85 ± 1.75 years in the Severe Group (*t* = 3.663, *P* < 0.001, Table 1). However, no significant differences were observed in BMI (kg/m²) between the two groups (15.35 ± 1.04 vs. 15.51 ± 0.95, *t* = 1.284, *P* = 0.200), nor in gender distribution (*χ*^2^ = 0.033, *P* = 0.856) or the presence of underlying diseases (*χ*^2^ = 0.247, *P* = 0.619). The imaging findings of the two patient groups are compared in Figure 1.

**Table 1:** Baseline characteristics of participants beween Non-severe group and severe group.

Parameters	Non-severe group (*n* = 195)	Severe group (*n* = 105)	t/*χ*^2^	*P*
Age (years)	6.65 ± 1.84	5.85 ± 1.75	3.663	<0.001
BMI, kg/m^2^	15.35 ± 1.04	15.51 ± 0.95	1.284	0.200
Gender, *n* (%)			0.033	0.856
Male	100 (51.28)	55 (52.38)		
Female	95 (48.72)	50 (47.62)		
Underlying diseases, *n* (%)			0.247	0.619
Yes	12 (6.15)	5 (4.76)		
No	183 (93.85)	100 (95.24)		

Tonsillar enlargement was significantly more prevalent in the severe group, affecting 35.24% compared to 18.46% in the non-severe group (*χ*^2^ = 10.433, *P* = 0.001) (Table 2). Tonsil enlargement was significantly more common in the Severe Group (35.24%) compared to the Non-severe Group (18.46%, *χ*^2^ = 10.433, *P* = 0.001). Similarly, conjunctival hyperemia was also more frequently observed in the Severe Group (14.29%) than in the Non-severe Group (7.18%, *χ*^2^ = 3.947, *P* = 0.047). The length of hospital stay (3.05 ± 1.24 days vs. 8.14 ± 2.12 days, *t* = 22.605, *P* < 0.001) and heating time (4.08 ± 0.26 days vs. 7.05 ± 1.13 days, *t* = 26.633, *P* < 0.001) were both significantly longer in the Severe Group. Additionally, lung wheezing (*χ*^2^ = 8.398, *P* = 0.004) and sore throat (*χ*^2^ = 4.155, *P* = 0.042) were more prevalent among severe cases. Conversely, there were no significant differences observed in cough duration (6.91 ± 1.85 days vs. 7.12 ± 1.86 days, *t* = 0.928, *P* = 0.354), temperature (37.25 ± 0.31°C vs. 37.35 ± 0.53 °C, *t* = 1.778, *P* = 0.078), presence of lung rales (89.74% vs. 85.71%, *χ*^2^ = 1.075, *P* = 0.300), muscle pain (4.10% vs. 9.52%, *χ*^2^ = 3.556, *P* = 0.059), and vomiting (24.62% vs. 21.90%, *χ*^2^ = 0.278, *P* = 0.598) between the two groups.

**Table 2:** Signs and symptoms between Non-severe group and severe group.

Parameters	Non-severe group (*n* = 195)	Severe group (*n* = 105)	t/*χ*^2^	*P*
Tonsil, *n* (%)			10.433	0.001
Enlargement	36 (18.46)	37 (35.24)		
Normal	159 (81.54)	68 (64.76)		
Conjunctiva, *n* (%)			3.947	0.047
Hyperemia	14 (7.18)	15 (14.29)		
Normal	181 (92.82)	94 (85.71)		
Length of stay, days	3.05 ± 1.24	8.14 ± 2.12	22.605	<0.001
Heating time, days	4.08 ± 0.26	7.05 ± 1.13	26.633	<0.001
Coughtime, days	6.91 ± 1.85	7.12 ± 1.86	0.928	0.354
Temperature, °C	37.25 ± 0.31	37.35 ± 0.53	1.778	0.078
Lung rales, *n* (%)	175 (89.74)	90 (85.71)	1.075	0.300
Lung wheezing, *n* (%)	23 (11.79)	26 (24.76)	8.398	0.004
Muscle pain, *n* (%)	8 (4.10)	10 (9.52)	3.556	0.059
Sore throat, *n* (%)	4 (2.05)	8 (7.62)	4.155	0.042
Vomitting, *n* (%)	48 (24.62)	23 (21.90)	0.278	0.598

The whole blood analysis comparison between the Non-severe Group and Severe Group of pediatric patients with adenovirus pneumonia revealed several significant differences (Table 3). PLT was significantly lower in the Severe Group compared to the Non-severe Group (*t* = 6.361, *P* < 0.001). Hemoglobin levels (Hb) were also slightly but significantly higher in the Severe Group than in the Non-severe Group (*t* = 2.379, *P* = 0.018). No significant differences were observed in WBC (7.35 ± 2.14 vs. 7.51 ± 1.79, *t* = 0.661, *P* = 0.509), lymphocyte percentage (8.99 ± 2.18 vs. 8.64 ± 2.58, *t* = 1.167, *P* = 0.245), lymphocyte count (860.12 ± 43.96 vs. 853.14 ± 46.14, t = 1.290, *P* = 0.198), or the percentage of neutrophils (*N*%, %) (45.27 ± 8.45 vs. 47.24 ± 8.32, *t* = 1.937, *P* = 0.054).

**Table 3:** Whole blood analysis comparison between non-severe and severe groups.

Parameters	Non-severe group (*n* = 195)	Severe group (*n* = 105)	t	*P*
PLT, ×10^9 ^L^−1^	295.41 ± 46.52	250.63 ± 63.55	6.361	<0.001
WBC, ×10^9 ^L^−1^	7.35 ± 2.14	7.51 ± 1.79	0.661	0.509
Lymphocyte, %	8.99 ± 2.18	8.64 ± 2.58	1.167	0.245
Lymphocyte count	860.12 ± 43.96	853.14 ± 46.14	1.290	0.198
Hb, g·L^−1^	105.46 ± 3.16	106.35 ± 2.94	2.379	0.018
*N*%, %	45.27 ± 8.45	47.24 ± 8.32	1.937	0.054

PLT, platelet; WBC, white blood cell; Hb, hemoglobin; N%, percentage of neutrophils.

Specifically, HBP levels were significantly higher in the Severe Group (46.12 ± 8.47) compared to the Non-severe Group (38.24 ± 4.52, *t* = 8.875, *P* < 0.001, Table 4). Similarly, TNF-α (2.61 ± 0.42 vs. 3.42 ± 1.07, *t* = 7.502, *P* < 0.001), IL-6 (3.98 ± 1.21 vs. 5.41 ± 1.42, *t* = 9.199, *P* < 0.001), and CRP levels (21.84 ± 3.25 vs. 25.08 ± 3.14, *t* = 8.333, *P* < 0.001) were all significantly elevated in the Severe Group. Conversely, there were no significant differences observed in PCT levels (0.52 ± 0.17 vs. 0.49 ± 0.16, *t* = 1.549, *P* = 0.123), LDH levels (379.65 ± 35.36 vs. 373.52 ± 36.28, *t* = 1.420, *P* = 0.157), complement component C3 levels (1.25 ± 0.24 vs. 1.22 ± 0.21, *t* = 1.154, *P* = 0.249), or complement component C4 levels (0.33 ± 0.12 vs. 0.32 ± 0.09, *t* = 0.318, *P* = 0.751) between the two groups.

**Table 4:** Comparison of erum parameters between Non-severe and severe groups.

Parameters	Non-severe group (*n* = 195)	Severe group (*n* = 105)	t	*P*
HBP (ng/ml)	38.24 ± 4.52	46.12 ± 8.47	8.875	<0.001
TNF-α (ng/ml)	2.61 ± 0.42	3.42 ± 1.07	7.502	<0.001
IL-6 (pg/ml)	3.98 ± 1.21	5.41 ± 1.42	9.199	<0.001
CRP (mg/L)	21.84 ± 3.25	25.08 ± 3.14	8.333	<0.001
PCT/ng·ml^−1^	0.52 ± 0.17	0.49 ± 0.16	1.549	0.123
LDH/U·L^−1^	379.65 ± 35.36	373.52 ± 36.28	1.420	0.157
C3 (g/L)	1.25 ± 0.24	1.22 ± 0.21	1.154	0.249
C4 (g/L)	0.33 ± 0.12	0.32 ± 0.09	0.318	0.751

HBP, heparin—binding protein; TNF—α, tumor necrosis factor—alpha; IL—6, Interleukin—6; CRP, C—reactive protein; PCT, procalcitonin; LDH, lactate dehydrogenase; C3, complement component 3; C4, complement component 4.

**Figure 2:**
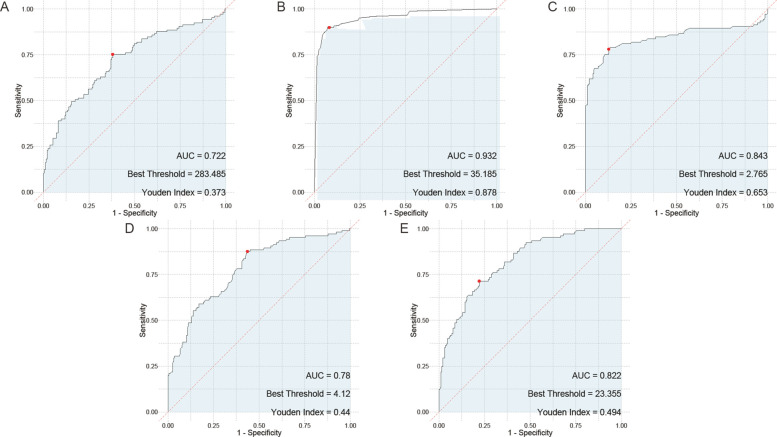
ROC curve of single serum HBP, TNF-α, IL-6, PLT, and CRP for the assessment of childhood adenovirus pneumonia. **(A)** ROC curve of PLT, **(B)** ROC curve of HBP, **(C)** ROC curve of TNF-α, **(D)** ROC curve of IL-6, **(E)** ROC curve of CRP. PLT, platelet; HBP, heparin—binding protein; TNF—α, tumor necrosis factor—alpha; IL—6, Interleukin—6; CRP, C—reactive protein.

In the assessment of childhood adenovirus pneumonia, the serum biomarkers including HBP, TNF-α, IL-6, PLT, and CRP were evaluated for their diagnostic performance through single marker ROC curve analysis. Among these biomarkers, HBP demonstrated the highest Area Under the Curve (AUC) value at 0.932, indicating its superior discriminative power. TNF-α also showed a significant AUC of 0.843, suggesting its effectiveness in distinguishing cases. Additionally, CRP had an AUC of 0.822, whereas IL-6 and PLT exhibited lower but notable AUC values of 0.78 and 0.722, respectively (Figure 2).

**Figure 3:**
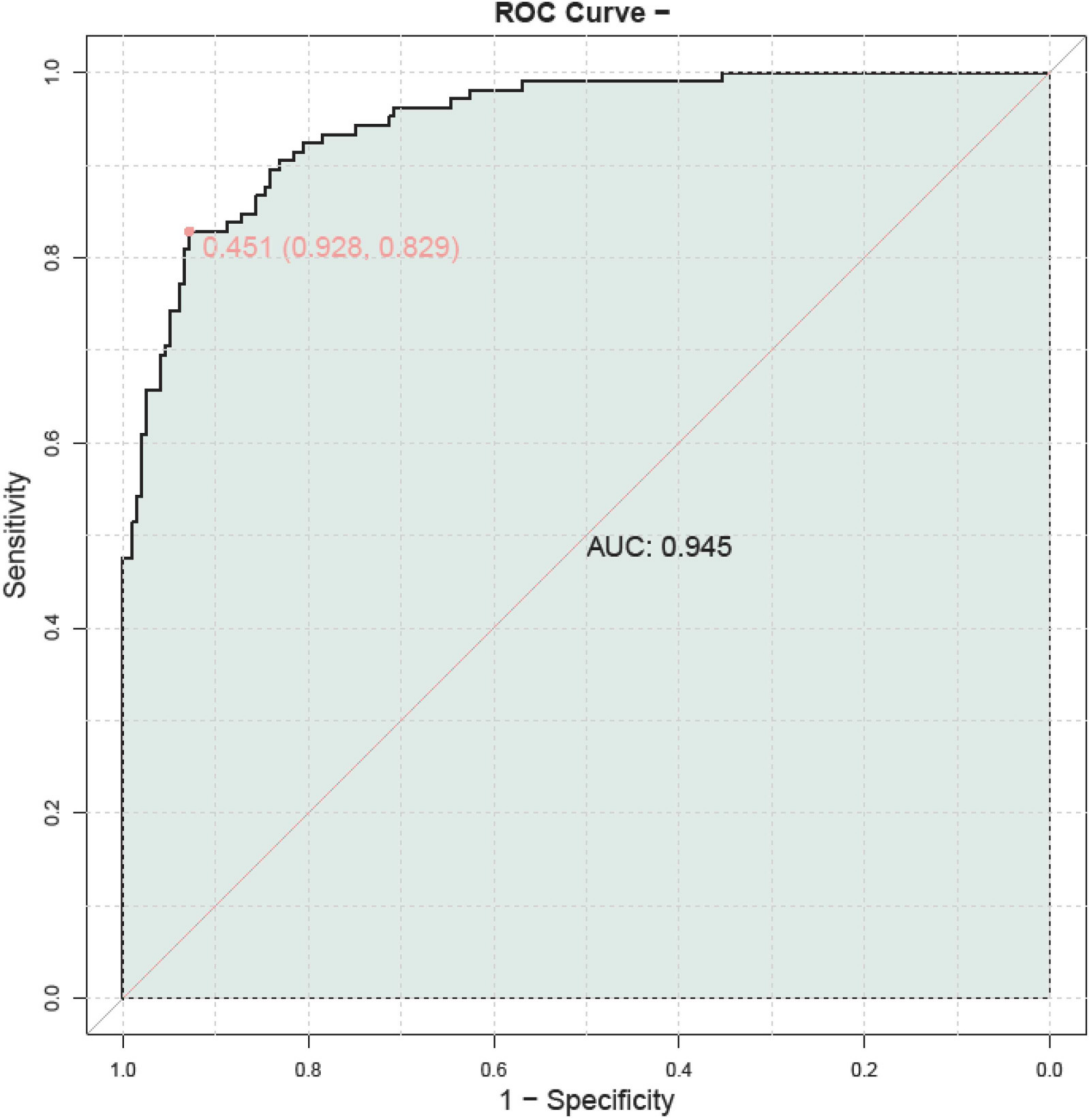
ROC curve of combined serum HBP, TNF-α, IL-6, PLT, and CRP for the assessment of childhood adenovirus pneumonia. PLT, platelet; HBP, heparin—binding protein; TNF—*α*, tumor necrosis factor—alpha; IL—6, Interleukin—6; CRP, C—reactive protein.

By combining serum HBP, TNF-α, IL-6, PLT, and CRP levels, we developed a composite predictive model for assessing the severity of adenovirus pneumonia in children. The model demonstrated a high predictive accuracy, with an AUC of 0.945, indicating its substantial prognostic value (Figure 3).

To exclude the influence of other inflammatory diseases on the inflammatory markers in the two groups of patients, we removed patients with inflammatory diseases for a supplementary analysis (Supplementary Tables S1–S4). The results showed a similar pattern (AUC = 0.964, Supplementary Figures S1, S2).

### 3.2 Post-treatment prognosis grouping

In our study examining the baseline characteristics between the Good Prognosis Group (*n* = 181) and the Poor Prognosis Group (*n* = 119, Table 5), significant differences were observed in age (*P* = 0.007) and BMI (*P* = 0.008). No significant differences were found in gender distribution (*χ*^2^ = 0.123, *P* = 0.726) or the presence of underlying diseases (*χ*^2^ = 0.411, *P* = 0.521) between the two groups. A significant difference was observed in the distribution of non-severe vs. severe children (*χ*^2^ = 6.558, *P* = 0.010).

**Table 5:** Baseline characteristics of children in good prognosis group and poor prognosis group.

Parameters	Good prognosis group (*n* = 181)	Poor prognosis group (*n* = 119)	t/*χ*^2^	*P*
Age (years)	6.45 ± 1.84	5.87 ± 1.77	2.708	0.007
BMI (kg/m^2^)	15.64 ± 1.22	15.26 ± 1.23	2.665	0.008
Gender [*n* (%)]			0.123	0.726
Male	86 (47.51%)	59 (49.58%)		
Female	95 (52.49%)	60 (50.42%)		
Underlying diseases			0.411	0.521
Yes	9 (4.97%)	8 (6.72%)		
No	172 (95.03%)	111 (93.28%)		
Non-severe Group/Severe Group	128 (70.72%)/53 (29.28%)	67 (56.3%)/52 (43.7%)	6.558	0.010

In the comparison of signs and symptoms between the Good Prognosis Group and the Poor Prognosis Group (Table 6), no significant differences were observed in most parameters examined, including tonsil enlargement (*χ*^2^ = 0.290, *P* = 0.590), conjunctiva hyperemia (*χ*^2^ = 0.360, *P* = 0.548), length of hospital stay (*P* = 0.289), heating time (*P* = 0.553), cough duration (*P* = 0.594), body temperature (*P* = 0.368), presence of lung rales (*χ*^2^ = 0.169, *P* = 0.681), lung wheezing (*χ*^2^ = 1.294, *P* = 0.255), muscle pain (*χ*^2^ = 0.183, *P* = 0.669), and vomiting (*χ*^2^=0.260, *P* = 0.610). However, sore throat showed a significant difference between groups (*χ*^2^ = 5.074, *P* = 0.024), with 3 children (1.66%) reported in the Good Prognosis Group compared to 9 children (7.56%) in the Poor Prognosis Group.

**Table 6:** Signs and symptoms of children in good prognosis group and poor prognosis group.

Parameters	Good prognosis group (*n* = 181)	Poor prognosis group (*n* = 119)	t/*χ*^2^	*P*
Tonsil, [*n* (%)]			0.290	0.590
Enlargement	46 (25.41%)	27 (22.69%)		
Normal	135 (74.59%)	92 (77.31%)		
Conjunctiva, [*n* (%)]			0.360	0.548
Hyperemia	19 (10.5%)	10 (8.4%)		
Normal	162 (89.5%)	109 (91.6%)		
Length of stay, days	5.35 ± 1.47	5.59 ± 2.12	1.062	0.289
Heating time, days	6.21 ± 2.25	6.36 ± 2.15	0.593	0.553
Coughtime, days	6.84 ± 2.33	6.99 ± 2.42	0.534	0.594
Temperature, °C	37.27 ± 0.34	37.31 ± 0.31	0.902	0.368
Lung rales, [*n* (%)]	161 (88.95%)	104 (87.39%)	0.169	0.681
Lung wheezing, [*n* (%)]	26 (14.36%)	23 (19.33%)	1.294	0.255
Muscle pain, [*n* (%)]	10 (5.52%)	8 (6.72%)	0.183	0.669
Sore throat, [*n* (%)]	3 (1.66%)	9 (7.56%)	5.074	0.024
Vomitting, [*n* (%)]	41 (22.65%)	30 (25.21%)	0.260	0.610

In the whole blood analysis comparison between the Good Prognosis Group and the Poor Prognosis Group (Table 7), significant differences were observed only in PLT counts. The PLT count was significantly higher in the Good Prognosis Group at 284.01 ± 32.37 × 10^9 ^L^−1^ compared to 257.41 ± 28.12 × 10^9 ^L^−1^ in the Poor Prognosis Group (*t* = 7.327, *P* < 0.001). No significant differences were found in WBC count (*P* = 0.565), lymphocyte percentage (*P* = 0.191), lymphocyte count (*P* = 0.552), Hb concentration (*P* = 0.836), or neutrophil percentage (*N*%, *P* = 0.623).

**Table 7:** Whole blood analysis comparison of children in good prognosis and poor prognosis groups.

Parameters	Good prognosis group (*n* = 181)	Poor prognosis group (*n* = 119)	t	*P*
PLT, ×10^9 ^L^−1^	284.01 ± 32.37	257.41 ± 28.12	7.327	<0.001
WBC, ×10^9 ^L^−1^	7.39 ± 2.41	7.54 ± 1.98	0.576	0.565
Lymphocyte, %	9.18 ± 4.97	8.52 ± 3.78	1.311	0.191
Lymphocyte count	859.51 ± 61.32	855.23 ± 60.36	0.595	0.552
Hb, g·L^−1^	105.59 ± 15.37	105.98 ± 16.94	0.207	0.836
*N*%, %	54.11 ± 8.72	54.63 ± 9.33	0.493	0.623

PLT, platelet; WBC, white blood cell; Hb, hemoglobin; *N*%, percentage of neutrophils.

In the serum analysis comparison between the Good Prognosis Group and the Poor Prognosis Group (Table 8), significant differences were observed in several inflammatory markers. Specifically, levels of HBP were significantly lower in the Good Prognosis Group at 41.22 ± 4.31 compared to 46.26 ± 4.27 in the Poor Prognosis Group (t = 9.951, *P* < 0.001). Similarly, TNF-α was lower in the Good Prognosis Group at 3.08 ± 0.39 vs. 3.51 ± 0.47 in the Poor Prognosis Group (*t* = 8.411, *P* < 0.001). IL-6 also showed a significant difference with levels of 4.38 ± 0.68 in the Good Prognosis Group compared to 5.06 ± 0.52 in the Poor Prognosis Group (t = 9.822, *P* < 0.001). CRP levels were slightly but significantly higher in the Poor Prognosis Group at 25.17 ± 2.62 compared to 23.77 ± 1.98 in the Good Prognosis Group (*t* = 4.963, *P* < 0.001). No significant differences were noted for PCT (*P* = 0.095), LDH (*P* = 0.353), C3 (*P* = 0.089), or C4 (*P* = 0.370).

**Table 8:** Serum analysis comparison of children in good prognosis and poor prognosis groups.

Parameters	Good prognosis group (*n* = 181)	Poor prognosis group (*n* = 119)	t	*P*
HBP (ng/ml)	41.22 ± 4.31	46.26 ± 4.27	9.951	<0.001
TNF-α (ng/ml)	3.08 ± 0.39	3.51 ± 0.47	8.411	<0.001
IL-6 (pg/ml)	4.38 ± 0.68	5.06 ± 0.52	9.822	<0.001
CRP (mg/L)	23.77 ± 1.98	25.17 ± 2.62	4.963	<0.001
PCT (ng·ml^−1^)	0.5 ± 0.15	0.52 ± 0.12	1.676	0.095
LDH (U·L^−1^)	373.36 ± 35.86	377.28 ± 35.33	0.931	0.353
C3 (g/L)	1.21 ± 0.26	1.26 ± 0.22	1.709	0.089
C4 (g/L)	0.31 ± 0.14	0.33 ± 0.13	0.898	0.370

HBP, heparin—binding protein; TNF—α, tumor necrosis factor—alpha; IL—6, Interleukin—6; CRP, C—reactive protein; PCT, procalcitonin; LDH, lactate dehydrogenase; C3, complement component 3; C4, complement component 4.

**Figure 4:**
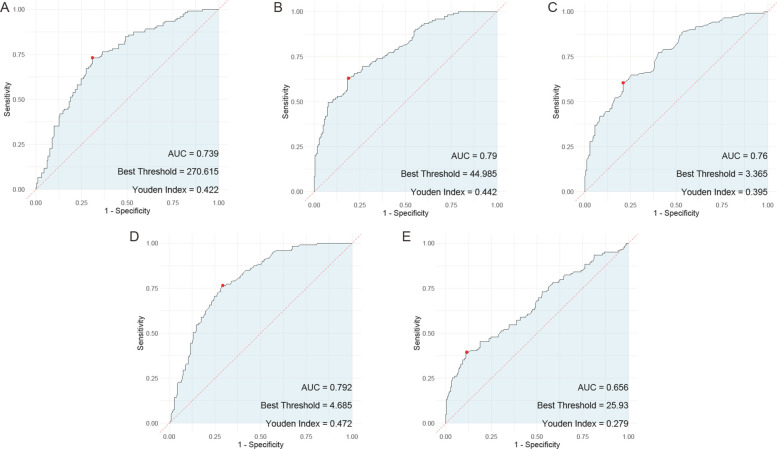
ROC curves of the predictive value of individual serum HBP, TNF-α, IL-6, PLT, and CRP tests for the prognosis of children with adenovirus pneumonia. **(A)** ROC curve of PLT; **(B)** ROC curve of HBP; **(C)**, ROC curve of TNF-α; **(D)**, ROC curve of IL-6; **(E)** ROC curve of CRP. PLT, platelet; HBP, heparin—binding protein; TNF—α, tumor necrosis factor—alpha; IL—6, Interleukin—6; CRP, C—reactive protein.

In the evaluation of individual serum biomarkers for prognosis of children with adenovirus pneumonia, ROC curve analysis was conducted to determine the predictive value of HBP, TNF-α, IL-6, PLT, and CRP. The results indicated that IL-6 had the highest Area Under the Curve (AUC) at 0.792, closely followed by both HBP and PLT, which exhibited AUC values of 0.79 and 0.739, respectively. TNF-α also showed a notable AUC of 0.76. In contrast, CRP demonstrated the lowest AUC among the markers evaluated, at 0.656 (Figure 4).

**Figure 5:**
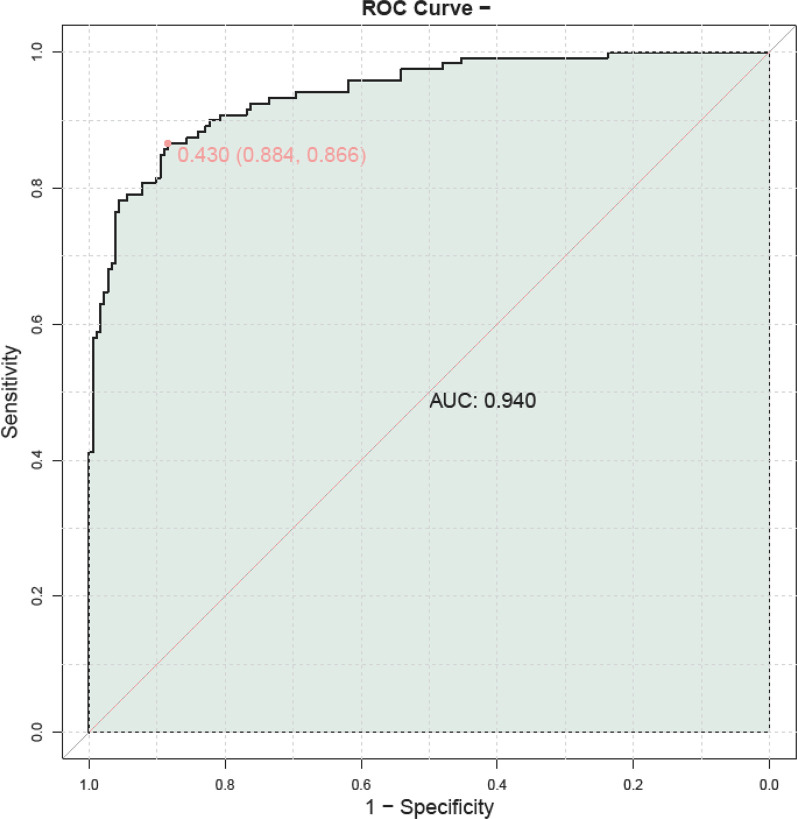
ROC curve illustrating the prognostic value of combined serum HBP, TNF-α, IL-6, PLT, and CRP in predicting outcomes for children with adenovirus pneumonia. PLT, platelet; HBP, heparin—binding protein; TNF—α, tumor necrosis factor—alpha; IL—6, Interleukin—6; CRP, C—reactive protein.

A prognostic model was constructed by combining serum HBP, TNF-α, IL-6, PLT, and CRP to predict outcomes in children with adenovirus pneumonia. The model demonstrated a high predictive value, with an AUC of 0.940, indicating its strong potential in forecasting patient prognosis (Figure 5).

Simultaneously, to validate the reliability of prognosis prediction and assess whether these parameters could predict outcomes within the severe and non-severe groups, we separately compared biomarkers in the severe group (Supplementary Tables S5–S8) and non-severe group (Supplementary Tables S9–S12) and constructed prognostic models for good vs. poor prognosis (Supplementary Figures S3–S6). The severe group comprised 53 patients with favorable prognosis and 52 with poor prognosis. The non-severe group included 128 patients achieving good prognosis vs. 67 children with adverse outcomes. The results demonstrated that the combined serum HBP, TNF-α, IL-6, PLT, and CRP exhibited strong predictive performance for patient prognosis in both severity subgroups, with AUC values of 0.960 (severe group) and 0.938 (non-severe group), respectively.

## 4 Discussion

The distinctive elevation of serum HBP, TNF-α, IL-6, and CRP levels in children with severe adenovirus pneumonia as well as those with poor prognosis underscored the central role of inflammatory pathways in the pathogenesis and prognosis of this infection. HBP, known for its ability to increase vascular permeability and contribute to the inflammatory response, emerges as a significant marker of acute inflammation and ensuing tissue injury in viral pneumonia (12, 20). Its notable elevation in severe children likely reflected an amplified systemic inflammatory response, correlating with disease severity and supporting its use as a prognostic biomarker (21).

The roles of TNF-α and IL-6 were likewise critical. These cytokines were cornerstone mediators in the inflammatory cascade, pivotal in the recruitment of immune cells and the propagation of inflammation (22). Their marked increase in severe children indicates a heightened inflammatory milieu, echoing previous research linking these cytokines to poor outcomes in various respiratory infections (23). TNF-α's influence on the immune response may exacerbate lung tissue damage, while IL-6 could potentially drive systemic inflammatory responses and acute-phase reactions, further compounding disease severity (24). Elevated HBP may promote the infiltration of inflammatory cells by enhancing vascular permeability, while the synergistic increase of TNF-α and IL-6 amplifies the local immune response. This “inflammatory storm” is directly related to lung tissue damage in critically ill children (25).

TNF—α and IL—6 play crucial roles in the crosstalk between the immune and coagulation systems. TNF—α can upregulate the expression of tissue factor (TF) on endothelial cells and monocytes. TF initiates the extrinsic coagulation pathway, leading to an increase in thrombin generation (26). This activation of the coagulation cascade can result in micro—thrombosis within the pulmonary vasculature, which further impairs gas exchange and contributes to lung injury (27). In addition, TNF—*α* can downregulate the expression of thrombomodulin, a protein that normally inhibits coagulation by activating protein C. This dual effect of TNF—α on promoting coagulation and inhibiting its regulation can lead to a pro—thrombotic state in severe adenovirus pneumonia (28).

CRP, an acute-phase protein, also significantly rises in response to inflammation. Its higher levels in severe children and those with poor prognosis pointed to its utility as a marker of systemic inflammation (29). Elevated CRP levels correlate with increased lung damage and systemic involvement, reflecting a robust host immune response (30). The synergy among these biomarkers highlights the overlapping and reinforcing pathways in the inflammatory response, which collectively may drive the outcomes to severe disease (31). A study by Haiqin Zhong found that in severe adenovirus pneumonia, the levels of CRP in children with severe adenovirus pneumonia were significantly higher than those in the mild adenovirus pneumonia group (32).

In this context, PLT revealed an inverse relationship with disease severity and prognosis (33). Thrombocytopenia in severe children may reflect platelet consumption and destruction within the inflammatory environment, associated with cytokine storms and disseminated intravascular coagulation (DIC) often seen in severe infections (34). The decrease in PLT among severe and poorer prognosis groups suggests not just its role as a passive biomarker but also as an active participant in the pathophysiological landscape of adenovirus pneumonia, with thrombocytopenia potentially serving as both a marker and mediator of severity.

The decrease in PLT was not only a consequence of consumption but also due to the direct and indirect effects of cytokines. As mentioned earlier, TNF—α and IL—6 can lead to a pro—thrombotic state, causing platelets to be trapped in micro—thrombi. Additionally, these cytokines can induce apoptosis in platelets. A study by Hantrakool et al. (35) demonstrated that TNF-α and IL-6 promote thrombosis by enhancing inflammation, endothelial dysfunction, platelet activation, and coagulation pathways. Furthermore, adenovirus infection may directly inhibit bone marrow hematopoiesis, leading to reduced platelet production and exacerbating thrombocytopenia (36). This direct suppression of hematopoietic stem cells by adenovirus has been observed in several viral infections and can significantly impact blood cell counts, including platelets (37). Moreover, adenovirus infection can trigger the production of autoantibodies that target platelet surface antigens, resulting in immune-mediated thrombocytopenia (38). The complex interplay among these biomarkers highlights the intricacy of immune and coagulation pathways underlying disease outcomes (39). The mediating role of cytokines like TNF-α and IL-6 in coagulation, fibrinolysis, and platelet function exemplifies the interconnectedness of immune responses and hemostasis in critical illness (40). This interrelation likely contributes to the observed distinct biomarker profiles between severity groups, emphasizing the multi-faceted nature of adenovirus pneumonia in children (19).

Our findings further underlined the age-related susceptibility in severe and poorer prognosis cases. Younger age groups, which showed higher prevalence in these categories, could reflect developmental differences in immune response, such as immature innate and adaptive immune functions, potentially impacting cytokine production and inflammatory responses (41, 42). This observation dovetailed with existing literature that associates younger age with poorer outcomes in respiratory infections.

Interestingly, while some traditional markers like WBC counts and other hematological parameters did not differentiate significantly between severity groups, the combination of HBP, TNF-α, IL-6, PLT, and CRP provided superior predictive accuracy. This supports the notion that pivotal insights lie within specific pathways rather than broad hematological shifts, pointing toward tailored biomarker panels for more refined clinical assessments (43).

Our composite prognostic model, achieving a high AUC for predictive accuracy, reinforced the clinical applicability of utilizing a multi-biomarker approach. This model had potential not only for stratifying risk but also for monitoring treatment response and guiding therapeutic interventions. Such approaches could refine patient management strategies, enabling targeted therapies for those at heightened risk and potentially improving outcomes through tailored interventions.

While our study provided valuable insights into the prognostic utility of combining serum HBP, TNF-α, IL-6, PLT, and CRP levels in children with adenovirus pneumonia, there were several limitations to consider. Our study sample size was relatively small and drawn from a single clinical center, which may limit the generalizability of the findings across broader populations. The study's cross—sectional nature prevented establishing causality between biomarker levels and disease outcomes. We can only observe associations at a single time point. The lack of longitudinal data may also restrict understanding of how these markers fluctuate throughout the disease course and post—recovery. Without following the patients over time, we cannot fully appreciate the dynamic nature of biomarker changes, which may be crucial for understanding the disease outcomes and recovery processes. Future research should address these limitations by incorporating larger, multi—center cohorts and longitudinal designs to validate and extend our findings. Furthermore, the combined use of multiple biomarkers may pose an economic burden on families due to the increased costs associated with additional testing and monitoring, which could limit the practical applicability of this approach in resource-limited settings. Future studies should explore ways to mitigate the economic impact on families while maintaining diagnostic accuracy.

In conclusion, this study demonstrated the relevant clinical value of combining serum HBP, TNF-α, IL-6, PLT, and CRP levels in the assessment and management of pediatric adenovirus pneumonia. To further enhance the diagnostic and evaluative value of our findings, we propose integrating multiple biomarkers into a comprehensive scoring system. The synergistic effects of HBP, TNF-α, IL-6, PLT, and CRP provide a more nuanced understanding of disease severity and outcomes compared to single markers alone. For instance, elevated levels of HBP and CRP in conjunction with increased TNF-α and IL-6 can serve as early indicators of severe disease, while thrombocytopenia may signal impending complications such as DIC. This multi-biomarker approach not only improves diagnostic accuracy but also facilitates timely intervention by identifying high-risk patients who may benefit from more aggressive treatment strategies. Moreover, the dynamic monitoring of these biomarkers over time could offer valuable insights into the disease course and response to therapy, enabling personalized and adaptive management plans for pediatric adenovirus pneumonia.

## Data Availability

The raw data supporting the conclusions of this article will be made available by the authors, without undue reservation.
