# Supplementary material for: The value of combined examination of serum serum heparin binding protein, tumor necrosis factor alpha, interleukin-6, platelet count, and c-reactive protein in evaluating the condition and prognosis of children with adenovirus pneumonia
Source: Front Pediatr. 2025 Sep 3;13:1620206. doi: 10.3389/fped.2025.1620206 (PMC12440316; doi:10.3389/fped.2025.1620206)
Supplement: Supplementary file 1 [file Datasheet1.docx]

Table S1: Baseline characteristics of participants in Non-severe Group and Severe Group without concurrent other inflammatory disease

| Parameters | Non-severe  Group(n=110) | Severe Group  (n = 58 ) | t/χ2 | P |
| --- | --- | --- | --- | --- |
| Age (years) | 6.41 ± 1.72 | 5.79 ± 1.62 | 2.267 | 0.025 |
| BMI, kg/m2 | 15.34 ± 1.05 | 15.54 ± 0.84 | 1.216 | 0.226 |
| Gender, n (%) |  |  | 0.288 | 0.591 |
| Male | 54 (49.09%) | 31 (53.45%) |  |  |
| Female | 56 (50.91%) | 27 (46.55%) |  |  |
| Underlying diseases, n (%) |  |  | 0 | 1 |
| Yes | 4 (3.64%) | 2(3.45%) |  |  |
| No | 106 (96.36%) | 56 (96.55%) |  |  |

Table S2: Signs and symptoms of participants in Non-severe Group and Severe Group without concurrent other inflammatory disease

| Parameters | Non-severe  Group(n=110) | | Severe Group  (n = 58 ) | | | t/χ2 | | P |
| --- | --- | --- | --- | --- | --- | --- | --- | --- |
| Tonsil, n (%) | |  | |  | 0.729 | | 0.393 | |
| Enlargement | | 17(15.45%) | | 12 (20.69%) |  | |  | |
| Normal | | 93 (84.55%) | | 46 (79.31%) |  | |  | |
| Conjunctiva, n (%) | |  | |  | 0 | | 1 | |
| Hyperemia | | 7(6.36%) | | 4(6.9%) |  | |  | |
| Normal | | 103 (93.64%) | | 54 (93.1%) |  | |  | |
| Length of stay, days | | 3.04 ± 1.12 | | 7.28 ± 2.08 | 14.434 | | < 0.001 | |
| Heating time, days | | 4.11 ± 0.25 | | 7.15 ± 1.08 | 21.112 | | < 0.001 | |
| Coughtime, days | | 6.87 ± 1.75 | | 6.84 ± 1.75 | 0.106 | | 0.916 | |
| Temperature, ℃ | | 37.14 ± 0.35 | | 37.19 ± 0.41 | 0.923 | | 0.357 | |
| Lung rales, n (%) | | 71 (64.55%) | | 41 (70.69%) | 0.645 | | 0.422 | |
| Lung wheezing, n (%) | | 11 (10%) | | 12 (20.69%) | 3.672 | | 0.055 | |
| Muscle pain, n (%) | | 5 (4.55%) | | 5 (8.62%) | 0.516 | | 0.472 | |
| Sore throat, n (%) | | 2 (1.82%) | | 3 (5.17%) | 0.546 | | 0.460 | |
| Vomitting, n (%) | | 24 (21.82%) | | 12 (20.69%) | 0.029 | | 0.865 | |

Table S3: Whole blood analysis of participants in Non-severe and Severe Groups without concurrent other inflammatory disease

| Parameters | Non-severe  Group(n=195) | Severe Group (n = 105) | t | P |
| --- | --- | --- | --- | --- |
| PLT, × 109 L−1 | 294.36 ± 45.85 | 247.65 ± 61.25 | 5.102 | < 0.001 |
| WBC, × 109 L−1 | 7.28 ± 2.16 | 7.42 ± 1.65 | 0.466 | 0.642 |
| Lymphocyte, % | 8.85 ± 2.07 | 8.56 ± 2.45 | 0.808 | 0.420 |
| Lymphocyte count | 852.54 ± 42.86 | 851.26 ± 45.23 | 0.181 | 0.857 |
| Hb, g·L−1 | 104.26 ± 3.24 | 105.24 ± 2.84 | 1.933 | 0.055 |
| N%, % | 45.12 ± 8.26 | 46.25 ± 8.65 | 0.834 | 0.405 |

PLT: Platelet, WBC: White Blood Cell, Hb: Hemoglobin, N%: Percentage of neutrophils

Table S4: Serum parameters of participants in Non-severe and Severe Groups without concurrent other inflammatory disease

| Parameters | Non-severe  Group(n=195) | Severe Group (n = 105) | t | P |  |
| --- | --- | --- | --- | --- | --- |
| HBP(ng/mL) | 38.12 ± 4.41 | 44.76 ± 7.85 | 5.955 | < 0.001 |  |
| TNF-α(ng/mL) | 2.51 ± 0.32 | 3.24 ± 0.85 | 6.256 | < 0.001 |  |
| IL-6(pg/mL) | 3.86 ± 1.15 | 5.36 ± 1.36 | 7.54 | < 0.001 |  |
| CRP (mg/L) | | 21.74 ± 3.17 | 24.46 ± 3.06 | 5.355 | < 0.001 |
| PCT /ng·mL−1 | | 0.48 ± 0.14 | 0.46 ± 0.14 | 0.833 | 0.406 |
| LDH /U·L−1 | | 378.45 ± 35.26 | 372.54 ± 35.74 | 1.027 | 0.306 |
| C3 (g/L) | | 1.16 ± 0.28 | 1.15 ± 0.15 | 0.044 | 0.965 |
| C4 (g/L) | | 0.29 ± 0.11 | 0.28 ± 0.08 | 0.586 | 0.559 |

HBP: Heparin - Binding Protein, TNF – α: Tumor Necrosis Factor – alpha, IL – 6: Interleukin – 6, CRP: C - Reactive Protein, PCT: Procalcitonin, LDH: Lactate Dehydrogenase, C3: Complement Component 3, C4: Complement Component 4

Figure S1: ROC curves of single serum HBP, TNF-α, IL-6, PLT, and CRP for assessing the severity of adenovirus pneumonia in children without other inflammatory diseases.

A :ROC curve of PLT, B: ROC curve of HBP, C: ROC curve of TNF-α, D: ROC curve of IL-6, D: ROC curve of CRP.

PLT: Platelet, HBP: Heparin - Binding Protein, TNF – α: Tumor Necrosis Factor – alpha, IL – 6: Interleukin – 6, CRP: C - Reactive Protein

Figure S2: ROC curve of combined serum HBP, TNF-α, IL-6, PLT, and CRP for assessing the severity of adenovirus pneumonia in children without other inflammatory diseases.

PLT: Platelet, HBP: Heparin - Binding Protein, TNF – α: Tumor Necrosis Factor – alpha, IL – 6: Interleukin – 6, CRP: C - Reactive Protein

Table S5: Comparison of baseline characteristics between the good prognosis group and poor prognosis group in severe patients.

| Parameters | Good Prognosis Group（n=53） | Poor Prognosis Group (n = 52) | t/χ2 | P |
| --- | --- | --- | --- | --- |
| Age (years) | 6.02 ± 1.45 | 5.49 ± 1.54 | 1.823 | 0.071 |
| BMI (kg/m2) | 15.82 ± 1.24 | 15.41 ± 1.23 | 1.710 | 0.090 |
| Gender [n (%)] |  |  | 0.079 | 0.778 |
| Male | 22(41.51%) | 23(44.23%) |  |  |
| Female | 31 (58.49%) | 29 (55.77%) |  |  |
| Underlying diseases, n (%) |  |  | 0 | 0.983 |
| Yes | 2 (3.77%) | 3 (5.77%) |  |  |
| No | 51 (96.23%) | 49 (94.23%) |  |  |

Table S6: Signs and symptoms between Good Prognosis Group and Poor Prognosis Group in severe patients

| Parameters | Good Prognosis Group（n=53） | Poor Prognosis Group (n = 52) | t/χ2 | | P |
| --- | --- | --- | --- | --- | --- |
| Tonsil, [n (%)] |  |  | 3.121 | 0.077 | |
| Enlargement | 23(43.4%) | 14 (26.92%) |  |  | |
| Normal | 30 (56.6%) | 38 (73.08%) |  |  | |
| Conjunctiva, [n (%)] |  |  | 1.835 | 0.176 | |
| Hyperemia | 10(18.87%) | 5 (9.62%) |  |  | |
| Normal | 43 (81.13%) | 47 (90.38%) |  |  | |
| Length of stay, days | 7.75 ± 1.47 | 8.39 ± 2.12 | 1.787 | 0.077 | |
| Heating time, days | 6.94 ± 2.11 | 7.26 ± 2.16 | 0.784 | 0.435 | |
| Coughtime, days | 6.82 ± 2.29 | 7.19 ± 2.43 | 0.796 | 0.428 | |
| Temperature, ℃ | 37.33 ± 0.27 | 37.37 ± 0.31 | 0.776 | 0.440 | |
| Lung rales, [n (%)] | 44 (83.02%) | 46 (88.46%) | 0.635 | 0.426 | |
| Lung wheezing, [n (%)] | 11 (20.75%) | 15 (28.85%) | 0.922 | 0.337 | |
| Muscle pain, [n (%)] | 4 (7.55%) | 6 (11.54%) | 0.133 | 0.716 | |
| Sore throat, [n (%)] | 2 (3.77%) | 6 (11.54%) | 1.281 | 0.258 | |
| Vomitting, [n (%)] | 10 (18.87%) | 13 (25%) | 0.577 | 0.448 | |

Table S7: Whole Blood Analysis Comparison Between Good Prognosis and Poor Prognosis Groups in severe patients

| Parameters | Good Prognosis Group（n=53） | Poor Prognosis Group (n = 52) | t | P |
| --- | --- | --- | --- | --- |
| PLT, × 109 L−1 | 258.64 ± 34.26 | 238.15 ± 29.18 | 3.295 | 0.001 |
| WBC, × 109 L−1 | 7.45 ± 2.41 | 7.63 ± 1.98 | 0.414 | 0.680 |
| Lymphocyte, % | 8.98 ± 2.97 | 8.23 ± 2.78 | 1.334 | 0.185 |
| Lymphocyte count | 857.51 ± 41.57 | 851.25 ± 41.62 | 0.771 | 0.442 |
| Hb, g·L−1 | 106.19 ± 15.69 | 106.31 ± 15.71 | 0.039 | 0.969 |
| N%, % | 57.15 ± 7.15 | 57.46 ± 8.39 | 0.203 | 0.839 |

PLT: Platelet, WBC: White Blood Cell, Hb: Hemoglobin, N%: Percentage of neutrophils

Table S8: Serum Analysis Comparison Between Good Prognosis and Poor Prognosis Groups in severe patients

| Parameters | Good Prognosis Group（n=53） | Poor Prognosis Group (n = 52) | | t | P |  |
| --- | --- | --- | --- | --- | --- | --- |
| HBP(ng/mL) | 44.72 ± 4.25 | 50.26 ± 5.25 | 5.948 | | < 0.001 |  |
| TNF-α(ng/mL) | 3.45 ± 0.59 | 4.11 ± 0.44 | 6.504 | | < 0.001 |  |
| IL-6(pg/mL) | 5.12 ± 0.91 | 5.69 ± 0.86 | 3.279 | | 0.001 |  |
| CRP (mg/L) | | 23.87 ± 1.94 | 26.27 ± 2.17 | 5.959 | | < 0.001 |
| PCT (ng·mL−1) | | 0.48 ± 0.17 | 0.52 ± 0.14 | 1.293 | | 0.199 |
| LDH (U·L−1) | | 371.65 ± 33.86 | 376.51 ± 35.12 | 0.723 | | 0.471 |
| C3 (g/L) | | 1.17 ± 0.27 | 1.24 ± 0.24 | 1.426 | | 0.157 |
| C4 (g/L) | | 0.31 ± 0.15 | 0.34 ± 0.13 | 0.937 | | 0.351 |

HBP: Heparin - Binding Protein, TNF – α: Tumor Necrosis Factor – alpha, IL – 6: Interleukin – 6, CRP: C - Reactive Protein, PCT: Procalcitonin, LDH: Lactate Dehydrogenase, C3: Complement Component 3, C4: Complement Component 4

Figure S3: ROC curves of the predictive value of individual serum HBP, TNF-α, IL-6, PLT, and CRP tests for the prognosis of children with severe adenovirus pneumonia

A :ROC curve of PLT, B: ROC curve of HBP, C: ROC curve of TNF-α, D: ROC curve of IL-6, D: ROC curve of CRP.

PLT: Platelet, HBP: Heparin - Binding Protein, TNF – α: Tumor Necrosis Factor – alpha, IL – 6: Interleukin – 6, CRP: C - Reactive Protein

Figure S4: ROC curve illustrating the prognostic value of combined serum HBP, TNF-α, IL-6, PLT, and CRP in predicting outcomes for children with severe adenovirus pneumonia.

PLT: Platelet, HBP: Heparin - Binding Protein, TNF – α: Tumor Necrosis Factor – alpha, IL – 6: Interleukin – 6, CRP: C - Reactive Protein

Table S9: Comparison of baseline characteristics between the good prognosis group and poor prognosis group in non-severe patients.

| Parameters | Good Prognosis Group（n=128） | Poor Prognosis Group (n = 67) | t/χ2 | P |
| --- | --- | --- | --- | --- |
| Age (years) | 6.91 ± 1.84 | 6.34 ± 1.77 | 2.077 | 0.039 |
| BMI (kg/m2) | 15.51 ± 1.22 | 15.06 ± 1.23 | 2.464 | 0.015 |
| Gender [n (%)] |  |  | 0.245 | 0.621 |
| Male | 64(50%) | 36(53.73%) |  |  |
| Female | 64 (50%) | 31 (46.27%) |  |  |
| Underlying diseases, n (%) |  |  | 0.056 | 0.813 |
| Yes | 7(5.47%) | 5(7.46%) |  |  |
| No | 121 (94.53%) | 62 (92.54%) |  |  |

Table S10: Signs and symptoms Comparison between Good Prognosis Group and Poor Prognosis Group in non-severe patients

| Parameters | Good Prognosis Group（n=128） | Poor Prognosis Group (n = 67) | t/χ2 | | P |
| --- | --- | --- | --- | --- | --- |
| Tonsil, [n (%)] |  |  | 0.060 | 0.806 | |
| Enlargement | 23(17.97%) | 13(19.4%) |  |  | |
| Normal | 105 (82.03%) | 54 (80.6%) |  |  | |
| Conjunctiva, [n (%)] |  |  | 0 | 1 | |
| Hyperemia | 9 (7.03%) | 5(7.46%) |  |  | |
| Normal | 119 (92.97%) | 62 (92.54%) |  |  | |
| Length of stay, days | 2.95 ± 0.47 | 3.09 ± 0.57 | 1.889 | 0.060 | |
| Heating time, days | 3.97 ± 1.25 | 4.11 ± 1.04 | 0.796 | 0.427 | |
| Coughtime, days | 6.85 ± 2.01 | 6.97 ± 1.94 | 0.416 | 0.678 | |
| Temperature, ℃ | 37.22 ± 0.35 | 37.27 ± 0.45 | 0.702 | 0.484 | |
| Lung rales, [n (%)] | 117 (91.41%) | 58 (86.57%) | 1.119 | 0.290 | |
| Lung wheezing, [n (%)] | 15 (11.72%) | 8 (11.94%) | 0.002 | 0.964 | |
| Muscle pain, [n (%)] | 6 (4.69%) | 2 (2.99%) | 0.036 | 0.850 | |
| Sore throat, [n (%)] | 1 (0.78%) | 3 (4.48%) | 1.434 | 0.231 | |
| Vomitting, [n (%)] | 31 (24.22%) | 17 (25.37%) | 0.032 | 0.859 | |

Table S11: Whole Blood Analysis Comparison Between Good Prognosis and Poor Prognosis Groups in non-severe patients

| Parameters | Good Prognosis Group（n=128） | Poor Prognosis Group (n = 67) | t | P |
| --- | --- | --- | --- | --- |
| PLT, × 109 L^−1^ | 314.21 ± 35.37 | 290.12 ± 29.44 | 4.774 | < 0.001 |
| WBC, × 109 L^−1^ | 7.21 ± 2.06 | 7.42 ± 1.98 | 0.682 | 0.496 |
| Lymphocyte, % | 9.58 ± 2.04 | 8.71 ± 2.18 | 2.762 | 0.006 |
| Lymphocyte count | 860.81 ± 41.85 | 859.23 ± 40.1 | 0.254 | 0.800 |
| Hb, g·L^−1^ | 104.59 ± 5.37 | 105.78 ± 4.92 | 1.509 | 0.133 |
| N%, % | 44.97 ± 8.72 | 45.62 ± 9.31 | 0.486 | 0.627 |

PLT: Platelet, WBC: White Blood Cell, Hb: Hemoglobin, N%: Percentage of neutrophils

Table S12: Serum Analysis Comparison Between Good Prognosis and Poor Prognosis Groups in non-severe patients

| Parameters | Good Prognosis Group（n=128） | Poor Prognosis Group (n = 67) | | t | P |  |
| --- | --- | --- | --- | --- | --- | --- |
| HBP(ng/mL) | 36.27 ± 4.36 | 41.48 ± 5.41 | 6.811 | | < 0.001 |  |
| TNF-α(ng/mL) | 2.47 ± 0.49 | 3.01 ± 0.47 | 7.305 | | < 0.001 |  |
| IL-6(pg/mL) | 3.88 ± 0.47 | 4.26 ± 0.42 | 5.544 | | < 0.001 |  |
| CRP (mg/L) | | 20.87 ± 1.95 | 21.97 ± 1.62 | 3.985 | | < 0.001 |
| PCT (ng·mL−1) | | 0.51 ± 0.14 | 0.53 ± 0.12 | 1.073 | | 0.285 |
| LDH (U·L−1) | | 374.36 ± 35.36 | 382.28 ± 35.34 | 1.486 | | 0.139 |
| C3 (g/L) | | 1.22 ± 0.31 | 1.27 ± 0.22 | 1.16 | | 0.248 |
| C4 (g/L) | | 0.29 ± 0.11 | 0.33 ± 0.08 | 2.935 | | 0.004 |

HBP: Heparin - Binding Protein, TNF – α: Tumor Necrosis Factor – alpha, IL – 6: Interleukin – 6, CRP: C - Reactive Protein, PCT: Procalcitonin, LDH: Lactate Dehydrogenase, C3: Complement Component 3, C4: Complement Component 4

Figure S5: ROC curves of the predictive value of individual serum HBP, TNF-α, IL-6, PLT, and CRP tests for the prognosis of children with non-severe adenovirus pneumonia

A :ROC curve of PLT, B: ROC curve of HBP, C: ROC curve of TNF-α, D: ROC curve of IL-6, D: ROC curve of CRP.

PLT: Platelet, HBP: Heparin - Binding Protein, TNF – α: Tumor Necrosis Factor – alpha, IL – 6: Interleukin – 6, CRP: C - Reactive Protein

Figure S6: ROC curve illustrating the prognostic value of combined serum HBP, TNF-α, IL-6, PLT, and CRP in predicting outcomes for children with non-severe adenovirus pneumonia.

PLT: Platelet, HBP: Heparin - Binding Protein, TNF – α: Tumor Necrosis Factor – alpha, IL – 6: Interleukin – 6, CRP: C - Reactive Protein
